# Nuclear microtubule filaments mediate non-linear directional motion of chromatin and promote DNA repair

**DOI:** 10.1038/s41467-018-05009-7

**Published:** 2018-07-02

**Authors:** Roxanne Oshidari, Jonathan Strecker, Daniel K. C. Chung, Karan J. Abraham, Janet N. Y. Chan, Christopher J. Damaren, Karim Mekhail

**Affiliations:** 1 Department of Laboratory Medicine and Pathobiology, MaRS Centre, University of Toronto, West Tower, 661 University Avenue, Toronto, ON M5G 1M1 Canada; 2 Department of Molecular Genetics, MaRS Centre, University of Toronto, West Tower, 661 University Avenue, Toronto, ON M5G 1M1 Canada; 3 Lunenfeld-Tanenbaum Research Institute, Mount Sinai Hospital, 600 University Avenue, Toronto, ON M5G 1X5 Canada; 4 Institute for Aerospace Studies, University of Toronto, 4925 Dufferin Street, Toronto, ON M3H 5T6 Canada; 5 Canada Research Chairs Program, University of Toronto, 1 King’s College Circle, Toronto, ON M5S 1A8 Canada; 6 Present Address: Broad Institute of MIT and Harvard, 75 Ames Street, Cambridge, MA 02142 USA

## Abstract

Damaged DNA shows increased mobility, which can promote interactions with repair-conducive nuclear pore complexes (NPCs). This apparently random mobility is paradoxically abrogated upon disruption of microtubules or kinesins, factors that typically cooperate to mediate the directional movement of macromolecules. Here, we resolve this paradox by uncovering DNA damage-inducible intranuclear microtubule filaments (DIMs) that mobilize damaged DNA and promote repair. Upon DNA damage, relief of centromeric constraint induces DIMs that cooperate with the Rad9 DNA damage response mediator and Kar3 kinesin motor to capture DNA lesions, which then linearly move along dynamic DIMs. Decreasing and hyper-inducing DIMs respectively abrogates and hyper-activates repair. Accounting for DIM dynamics across cell populations by measuring directional changes of damaged DNA reveals that it exhibits increased non-linear directional behavior in nuclear space. Abrogation of DIM-dependent processes or repair-promoting factors decreases directional behavior. Thus, inducible and dynamic nuclear microtubule filaments directionally mobilize damaged DNA and promote repair.

## Abstract (web summary)

Following DNA damage, different processes come to action to aid repair. The authors here find that microtubule filaments within the cell nucleus capture and non-randomly mobilize damaged chromatin to mediate DNA repair.

## Introduction

In eukaryotic cells, genetic information is encoded in DNA molecules inside the nucleus, which is defined by the nuclear envelope^1^. DNA wraps around core histone proteins constituting chromatin, the compaction of which creates chromosomes that collectively form an ordered spatial genome organization during the interphase stage of the cell cycle. This organization shares many principles and regulatory factors across eukaryotes^1–3^.

Inside nuclei, spatial genome organization is not static but dynamically responds to various endogenous or exogenous cues. One striking example of the non-static nature of eukaryotic genomes is highlighted by studies focusing on the connections between the DNA damage response and genome organization. Damaged DNA exhibits various degrees of increased mobility in yeast, fly, worm, mouse, and human nuclei^4–22^. Mutations compromising this increased mobility abrogate repair^4–22^. Collectively, these studies reveal that the increased mobility of damaged DNA supports repair by moving damage to repair-conducive nuclear neighborhoods such as nuclear pore complexes (NPCs), clustering damaged DNA loci, facilitating contacts between damaged and intact DNA, or relocating damage outside of heterochromatin^4–22^.

Mobility and repair of various yeast and mammalian damaged DNA loci is compromised upon disruption of microtubules and kinesin motors^4,6,22–24^. In budding yeast, DSBs repairable by the homologous recombination subtype break-induced replication (BIR) are re-localized to the NPC sub-complex NUP84, which encompasses seven subunits including the Nup84 and Nup145c proteins^4,15,25–27^. Efficient mobility and repair of these BIR-repairable DSBs require the microtubule-stabilizing α-Tubulin isoform Tub3 and the evolutionarily conserved motor protein complex Kinesin-14, whose catalytic subunit is the Kar3 protein^4^. Repair also depends on the Rad9 DNA damage response mediator, Rad52 homologous recombination protein, and Pol32 BIR factor^4,7,8,26^. In contrast, the disruption of actin polymerization is ineffectual in this setting^22^. How microtubules and motors mobilize damaged DNA to promote repair in yeast remains unclear.

Studies in murine and fission yeast cells suggest that the linker of nucleoskeleton and cytoskeleton complex may bridge the nuclear envelope, relaying cytoplasmic forces onto damaged DNA resulting in its mobilization inside the nucleus^6,28–30^. However, kinesin motors are physically enriched at damaged DNA sites inside the nucleus in both budding yeast and mammalian cells, suggesting that molecular motors cannot be transporting damaged DNA inside nuclei by moving along cytoplasmic microtubules^4,24^. Moreover, the roles of microtubules and molecular motors in DNA repair are not separate since genetic studies reveal that the disruption of motors and microtubules is epistatic in terms of compromising DNA repair^4,6^. Therefore, how microtubules and motors mobilize damaged DNA to promote repair cannot be explained by existing models and remains unclear.

In addition, microtubules and motors typically mediate the directional motion of cargo. In stark contrast, studying the mobility of damaged DNA whose repair is dependent on microtubules and motors using a type of single particle motion analysis called mean square displacement (MSD) has paradoxically revealed random diffusive mobility^4,6^. This damaged DNA mobility paradox is observed in both yeast and mammalian cells. Thus, we reasoned that specific yet unclear cooperation between cytoplasmic microtubules and nuclear molecular motors may mobilize damaged DNA in the nucleus. Such cooperation could result in the non-directed motion of damaged DNA. Alternatively, microtubules and motors could be exerting directed transport that is masked by potentially confounding variables.

Here, we reveal and characterize DNA damage-inducible intranuclear microtubule filaments (DIMs) and show that they can capture and mobilize damaged DNA inside the nucleus via cooperation with Kar3 and Rad9. We report that genetic alterations that compromise or further promote DIM formation abrogate and hyper-activate DNA repair, respectively. Using live single-cell super-resolution imaging, we found that damaged DNA linearly moves along DIMs. However, the DIMs themselves are not static and oscillate inside the nucleus. By utilizing a new analytical approach that can compensate for DIM movements, we also reveal that damaged DNA exhibits increased non-linear directional behavior in nuclear space across cell populations. Overall, by identifying specific roles for nuclear microtubules and related factors in damaged DNA mobility-dependent repair, our work resolves the damaged DNA mobility paradox while widely impacting our understanding of genome organization, chromatin motion, and cell survival.

## Results

### DNA damage-inducible nuclear microtubules and its modulators

**Fig. 1:**
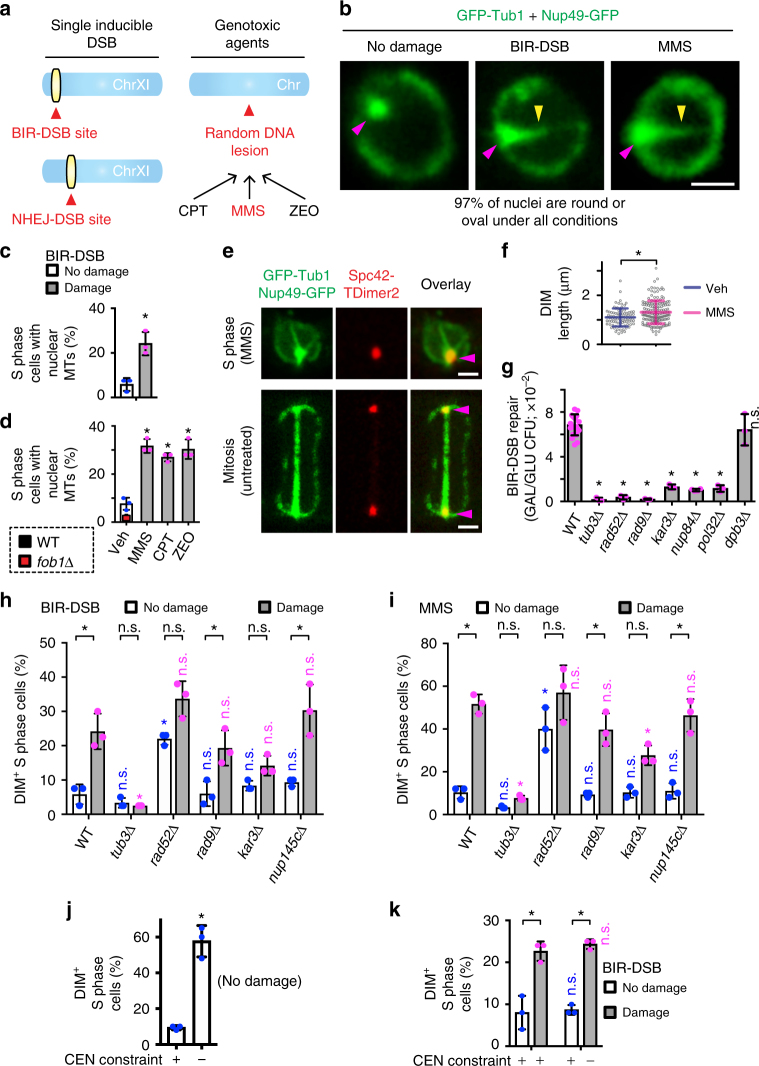
Identification of DIMs and its modulators. **a** Systems used to induce DNA damage. **b** Confocal imaging of nuclei in cells under non-damaging and DNA-damaging conditions. Arrowheads, MTOC (magenta) and microtubule filaments (yellow). **c**, **d** Intranuclear microtubule filament quantitation via confocal microscopy. Veh vehicle. **e** Confocal imaging showing DIM emanating from an S-phase cell MTOC and differing from the mitotic spindle. **f** DIM length measurements. **g** Quantification of BIR-DSB repair. WT wild type. **h**, **i** Effect of different gene knockouts on DIM levels. Wild-type cells in (**c**, **h**) are the same. **j** Effect of relieving centromeric constraint on DIMs. **k** Effect of combining BIR-DSB induction with the relief of centromeric constraint. **b**, **e** Scale bar, 1 µm. **c**, **d**, **f**, **g** Mean ± s.d.; *N* = 3. *P ≤ 0.03, two-tailed unpaired *t* test. **h**–**k** Mean ± s.d.; *N* = 3. *P ≤ 0.015, two-way ANOVA Sidak’s multiple comparison test. n.s., not statistically significant. Statistical symbols in blue indicate a comparison to undamaged WT control, magenta to damaged WT control, and black compares undamaged to damaged cells for each genotype. Individual data points of undamaged (blue) and damaged (magenta) cells are shown

To test if DNA damage alters microtubule organization, we co-expressed the green fluorescent protein (GFP)-tagged α-Tubulin protein Tub1 and NPC subunit Nup49 in two experimental systems (Fig. 1a). In the first, we used a single galactose-inducible and BIR-repairable DSB near the end of chromosome XI (BIR-DSB)^4,26^. As control, we used a more internally located galactose-inducible but non-homologous end joining (NHEJ)-repairable DSB (NHEJ-DSB)^4,26^. Repair of the BIR-DSB, but not the NHEJ-DSB, is dependent on microtubules and molecular motors^4^. In the second system, we treated cells with DNA-damaging drugs. Specifically, we used the replication fork-stalling methyl methanesulfonate (MMS), the radiomimetic zeocin or the topoisomerase-I inhibitor camptothecin^7,31^.

Cells subjected to BIR-DSB, not NHEJ-DSB, exhibited elevated levels of nuclear microtubule filaments, which did not distort the majority of nuclei (Fig. 1b, c; Supplementary Fig. 1a, b). These nuclear microtubule filaments were also induced in cells treated with MMS, zeocin, or camptothecin (Fig. 1b, d). BIR-DSB and MMS induced preferentially one nuclear microtubule filament per cell, although more MMS-treated cells displayed two or three nuclear microtubule filaments (Supplementary Fig. 1c). These results are consistent with the ability of genotoxic drugs to damage more than one DNA locus, unlike our BIR-DSB system. Of note, cells not subjected to exogenous DNA damage exhibited low levels of nuclear microtubule filaments, possibly reflecting endogenous DNA damage events (Fig. 1d). Indeed, hyperstabilization of ribosomal DNA repeats, which constitute a major source of DNA replication-induced DNA damage, via deletion of the Fork block protein 1, greatly decreased nuclear microtubule filaments in vehicle-treated cells (Fig. 1d)^1,32–35^. These results suggest that nuclear microtubule filaments form in response to endogenous or exogenous DNA damage. Thus, we defined these filaments as DIMs. DIMs emanate from the microtubule-organizing center (MTOC), which we marked by fusing the Spc42 MTOC protein to tandem dimer mutant of DsRed (TDimer2) (Fig. 1e)^5^. However, unlike nascent and mature mitotic microtubule spindles, DIMs are monopolar and exhibit rapidly decreasing fluorescence intensity as the distance to the MTOC increases (Fig. 1e; Supplementary Fig. 1d, e). We also noted that the DIMs in cells not subjected to exogenous DNA damage are only slightly shorter than those in cells subjected to exogenous damage, suggesting that DIMs reflecting endogenous or exogenous DNA damage events are similar (Fig. 1f). Thus, DNA-damaging events can induce nuclear microtubule filaments, herein defined as DIMs.

Cells deficient in homologous recombination-based processes are hypersensitive to MMS treatment^31^. Similarly, efficient BIR/homologous recombination-based repair of the BIR-DSB requires Tub3, Rad52, Rad9, Nup84, and Kar3 (Fig. 1g)^4^. Therefore, we asked if these repair-promoting factors alter DIMs in cells subjected to BIR-DSB or MMS. In both cases, *TUB3* knockout (*tub3*Δ) suppressed DIMs following DNA damage induction (Fig. 1h, i). This result is important, as Tub3 is not essential for mitotic or meiotic spindle formation^36^. In addition, loss of Rad52 induced DIMs even before BIR-DSB activation or MMS treatment (Fig. 1h, i). In fact, upon damage induction, DIM levels were not further induced in *rad52Δ* cells (Fig. 1h, i). Similar results were obtained in cells lacking the Rad51 homologous recombination protein (Supplementary Fig. 1f). Additionally, the loss of Rad51 also partly compromised MMS-induced DIM formation (Supplementary Fig. 1f). Moreover, while the disruption of Rad9 or the NUP84 subunit Nup145c was ineffectual, *kar3Δ* partly decreased DIM levels (Fig. 1h, i). These findings indicate that DIMs are highly and partly dependent on Tub3 and Kar3, respectively. In the absence of exogenous DNA damage, Rad52 and Rad51 repress DIM formation, possibly via repair of endogenous DNA damage. Rad51 also partly contributes to DIM formation in the presence of MMS. Disruption of NPCs, or unexpectedly the DNA damage response, has no effect.

Anchorage of centromeres at the MTOC is a major physical restraint that is exerted onto chromosomes and limits the mobility of DSBs^5^. Thus, we asked if the loss of centromeric restraint at one chromosome is sufficient to trigger DIMs. Indeed, we found that relieving physical restraint on the centromere of chromosome III (CEN3) by forcing transcription through the centromere^5^ was sufficient to induce DIMs even without BIR-DSB induction (Fig. 1j). Further, the concomitant artificial relief of CEN3 constraint and BIR-DSB induction did not further induce DIMs (Fig. 1k). Thus, relief of centromeric constraint is epistatic with DSB induction in terms of triggering DIMs.

### DIMs capture/mobilize damaged DNA and promote repair

**Fig. 2:**
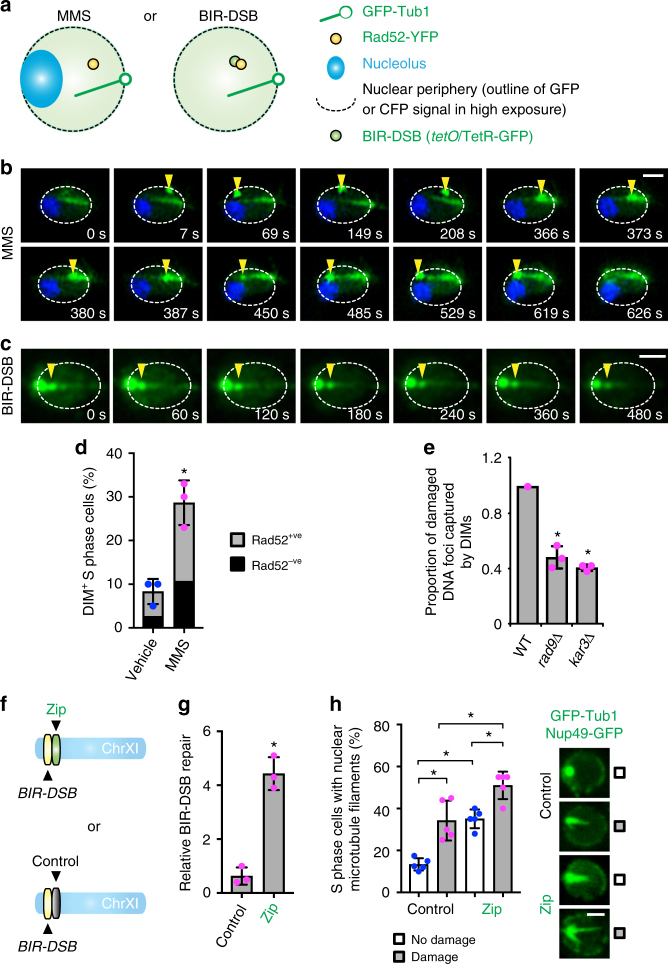
DIMs capture/mobilize damaged DNA and promote DNA repair. **a** Systems used to visualize damaged DNA in live wild-type cells. **b**, **c** Time-lapse super-resolution microscopy showing the dynamics of Rad52-marked damaged DNA (yellow arrowhead) relative to DIMs. Scale bar, 1 µm. **d** Quantification of the proportion of DIM-positive S-phase cells with and without Rad52 foci. Mean ± s.d.; *N* = 3. *P = 0.0039, two-tailed unpaired *t* test. **e** Relative effect of *rad9Δ* or *kar3Δ* on the ability of DIMs to capture damaged DNA. Relative mean ± s.d.; *N* = 3. *P ≤ 0.0011, two-tailed unpaired *t* test. **f** Zip or control DNA sequence integration near the BIR-DSB site. **g** Effect of the zip code on BIR-DSB repair. Mean ± s.d.; *N* = 3. *P = 0.0007, two-tailed unpaired *t* test. **h** Quantification and representative images from confocal microscopy showing the effect of the zip code on DIM levels in the absence or presence of BIR-DSB induction. Mean ± s.d.; *N* = 5. *P ≤ 0.0067, two-way ANOVA Sidak’s multiple comparison test. Scale bar, 1 µm. Individual data points of undamaged (blue) and damaged (magenta) cells are shown

Next, we aimed to co-visualize DIMs and damaged DNA. We used BIR-DSB or MMS in cells expressing fluorescently labeled Tub1 and Rad52, which marks the site of repair on damaged DNA (Fig. 2a; Supplementary Fig. 2a, b)^4,7^. We also visualized the nucleolus in MMS-treated cells using Nop1-CFP and observed the BIR-DSB site before damage induction using *tet* operator (*tetO*) sequences and fluorescent Tet repressor (Fig. 2a; Supplementary Fig. 2a, b)^37^. Upon MMS treatment or BIR-DSB induction, DIM-positive cells exhibited a Rad52 focus that roamed the nucleus or was captured by DIMs (Fig. 2b, c; Supplementary Fig. 2c, d). The captured Rad52 focus moved away from the MTOC along dynamic DIMs towards the nuclear periphery (Fig. 2b, c). In fact, we visualized the Rad52 focus getting captured by and moving along DIMs to the nuclear periphery before focus dissolution, which marks repair completion (Fig. 2b; Supplementary Movie 1)^38^. Consistent with the observation that DIM formation can precede Rad52 focus assembly, not all DIM-containing cells displayed a Rad52 focus (Fig. 2b, d). More importantly, loss of Rad9 or Kar3 abrogated the ability of DIMs to capture Rad52 foci (Fig. 2e). Therefore, DNA damage response and motor proteins allow DIMs to capture damaged DNA, which can then move along DIMs towards the nuclear periphery before repair focus dissolution. The DIM-mediated movement of damaged DNA is not perfectly linear within nuclear space since DIMs are themselves mobile.

We next asked if the hyper-induction of DIMs can hyperactivate DNA repair. We took advantage of the fact that eukaryotic genomes harbor regulated DNA sequences called zip codes, which promote interactions of their host DNA loci with NPCs^4,39,40^. Insertion of a truncated and thus constitutively active DNA zip code, but not a scrambled code control, near the BIR-DSB site is known to increase its interaction with Nup84 and hyper-activate repair (Fig. 2f, g)^4,39,40^. Since this zip code fails to target the BIR-DSB site to NPCs upon Kinesin-14 disruption^4^, we reasoned that the zip code might itself rely on nuclear microtubules. Indeed, in zip code-containing cells, intranuclear microtubule filaments were induced even before DSB induction (Fig. 2h). These elevated intranuclear microtubule filament levels were further increased following DSB induction indicating that the zip code and DSB promote nuclear microtubule filaments via at least partly independent processes (Fig. 2h). The scrambled code control failed to induce intranuclear microtubules before or after DSB induction and did not hyper-activate DNA repair (Fig. 2f–h). These findings reveal that the DNA zip code hyper-induces intranuclear microtubule filaments and hyper-activates DNA repair.

### Damage and Kar3 increase non-linear directionality of DNA

The mobility of damaged DNA across the cell population can be studied using MSD = <(*x*(*t* + Δ*t*) − *x*(*t*))^2^>, where *x* is the position of the damaged DNA and *t* is time^41^. Using MSD, one can also calculate the radius of confinement (*R*_c_), a useful value reflecting the subnuclear area explored by DSBs. In addition, curve fitting can be achieved using MSD *=* *Γt*^α^, where *Γ* is a generalized coefficient, to yield an *α* exponent coefficient reflecting the type of single particle mobility^42^. In this case, *α* ~ 1 reflects normal diffusion, *α* < 1 reflects subdiffusion or anomalous diffusion, and *α* ≥ 2 reflects directed mobility. Several studies employing MSD suggest that damage increases the ability of different DNA loci to explore a larger nuclear volume^4–8,22^. However, in contrast to our herein presented data so far, previous MSD-based analyses, which were conducted by other groups and ourselves, of DSB mobility across cell populations revealed normal or anomalous diffusion and not bona fide directed motion^4–8,43^. Nonetheless, one key report suggested that MSD analysis of DSB mobility in human cells can yield *α* ≥ 2 when DSBs are monitored for very long periods of time (e.g., 60 min) and the *α* coefficient is calculated using a subset of time points at which directed motion can be recognized by the human eye^42^. Thus, MSD analysis of DSB mobility tracking coordinates across cell populations reveals important mobility features but does not readily detect rare directional motions^44^.

**Fig. 3:**
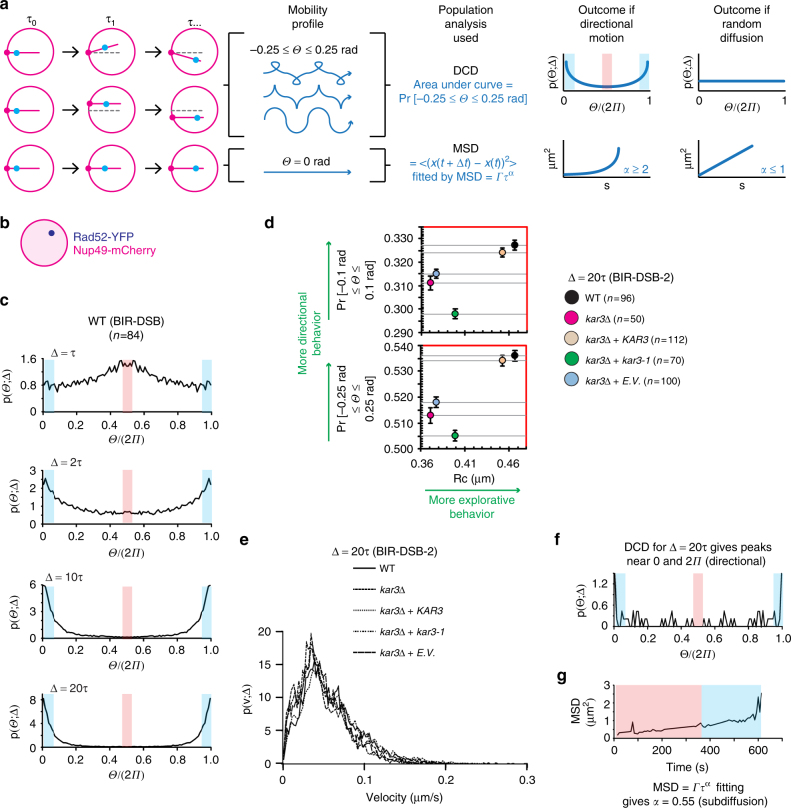
Damaged DNA exhibits non-linear directional motion in the nucleus. **a** DIM (magenta sticks) angle (*Θ*) deviations of up to 0.25 rad from a straight line can cause DSB single particles (blue) to exhibit non-linear directional motion detectable by DCD, not MSD. **b** System used in DCD analysis of BIR-DSBs. Rad52-YFP-marked damaged DNA was tracked relative to the perinuclear Nup49-mCherry. **c** DCD analysis of damaged DNA mobility reveals non-linear directionality. Shown are relative angle distributions with indicated temporal coarse grainings (Δ) for a single induced BIR-DSB. Histograms are for Rad52-YFP particles monitored for 3 min using 1.5 s long steps. **d** DCD-derived probabilities of BIR-DSB-2 moving with up to 0.1 or 0.25 rad angle deviation from a straight line were plotted relative to MSD-derived *R*_c_ values. Error bars, DCD probability errors. **e** DCD-derived distributions of the velocities of damaged DNA particles for cells shown in **d**. **f**, **g** Comparison of DCD (**f**) and MSD (**g**) analyses for a single damaged DNA particle shown in Fig. 2b and Supplementary Movie 1. Blue and red shading, respectively, highlight either (**f**) DCD peaks detecting directional and anti-directional behavior or (**g**) time windows during which the particle is known to exhibit directional or non-directional motion along DIMs in Supplementary Movie 1

In fact, we observed that damaged DNA only transiently moves along DIMs (Fig. 2b; Supplementary Movie 1). In addition, changes occur in the angle of DIMs emanating from the MTOC, which also moves along the nuclear periphery (Supplementary Fig. 2e, f). This results in up to ±0.25 rad (±14.4°) DIM angle deviations over time (Fig. 3a). Thus, we suspected that damaged DNA moving in the nucleus might exhibit non-linear yet directional motion (Fig. 3a). Therefore, we analyzed DSB mobility within nuclear space across the cell population by using directional change distribution (DCD)^4,5,44^. DCD assesses changes in the angle of a moving particle and encompasses a temporal coarse graining (Δ) that can be increased to reveal broader motion profiles (Fig. 3a)^44^. DCD using Δ = 1.5 s (1*τ*) revealed that a Rad52-tracked BIR-DSB monitored for 3 min commonly switches to the opposite direction (Π or 180°), the antithesis of directionality (Fig. 3b, c). Importantly, as Δ increased from 1 to 20*τ*, peaks emerged near 0Π and 2Π, the benchmarks for directed motion (Fig. 3c). We then asked if another BIR/Kinesin14-repairable DSB that is located on chromosome V (BIR-DSB-2) exhibits directionality (Supplementary Fig. 3a)^4^. Indeed, BIR-DSB-2 induction triggered DIMs and caused Rad52-YFP foci to exhibit directionality in DCD as Δ increased to 20*τ* (Supplementary Fig. 3b, c). Thus, consistent with our single-cell data, DCD analysis of damaged DNA across the cell population reveals a directional motion behavior.

Analysis of areas under the curve in a DCD histogram (Supplementary Fig. 3c), which is a probability density function, can be used to calculate the probability of an induced DSB to move with ±0.1 rad (±7.2°) or ±0.25 rad (±14.4°) deviation from a straight line (Fig. 3a). Thus, we plotted these DCD angle deviation probabilities at Δ = 20*τ* in relation to MSD analysis-derived mean *R*_c_ values in order to compare the impact of Kar3 modulation on both of the directional and explorative behaviors of damaged DNA. Importantly, *kar3Δ* partly yet significantly decreased both directional and explorative behaviors of an induced BIR-DSB-2 (Fig. 3d). The ATP hydrolysis-deficient Kar3 point mutant *kar3-1* is a so-called motor rigor mutant, which means that it binds microtubules but is motor-dead making it an impediment to microtubule sliding^4,45^. This mutant is also incapable of promoting BIR-DSB-2 repair^4^. In *kar3Δ* cells, introduction of Kar3 almost fully restored directional and explorative behaviors (Fig. 3d). In stark contrast, introduction of *kar3-1* into *kar3Δ* cells further decreased directional behavior despite slightly increasing exploration (Fig. 3d). This indicates that changes in directionality and subnuclear exploration are generally concordant but can be uncoupled. This also suggests that the more complete disruption of microtubule-based processes by the *kar3-1* mutant^4,45^ decreases the directed motion but increases the non-directed mobility of damaged DNA. Consistent with the importance of directionality for DNA repair, introduction of *kar3-1* in *kar3Δ* cells fails to increase BIR-DSB-2 repair efficiency^4^ despite increasing *R*_c_ values (Fig. 3d). Thus, DCD-based analysis of damaged DNA mobility across the cell population reveals non-linear directional behavior. This result is consistent with the DIM-mediated non-linear directed motion of damaged DNA that we observe at the single-cell level. Moreover, Kar3, which promotes DIMs and their ability to capture damaged DNA, increases the directional motion and subnuclear exploration behaviors of damaged DNA.

DCD can also be used to compare the normal distribution of velocities exhibited by damaged DNA across cell populations. No differences were observed between the velocity distributions of damaged DNA in wild-type, *kar3Δ*, *kar3Δ* + *KAR3*, and *Kar3Δ* + *kar3-1* cells (Fig. 3e). This indicates that directional and explorative behaviors are not necessarily coupled to changes in velocity.

Next, we aimed to directly compare the ability of MSD and DCD to detect directional behavior in a single ~600 s long movie encompassing ~200 s during which a Rad52 focus can actually be seen moving along DIMs (Fig. 2b; Supplementary movie 1). DCD using Δ = 20*τ* easily revealed peaks near 0Π and 2Π, the benchmarks for directionality (Fig. 3f). In stark contrast, MSD analysis of the same Rad52 focus revealed an α coefficient of 0.55, which would suggest sub-diffusive and not directed motion. Thus, while MSD yields critical information such as *R*_c_ values, DCD better recognizes transient and non-linear directional behaviors both on the cell population and single-cell levels.

### Damage increases inherent non-linear directionality of DNA

**Fig. 4:**
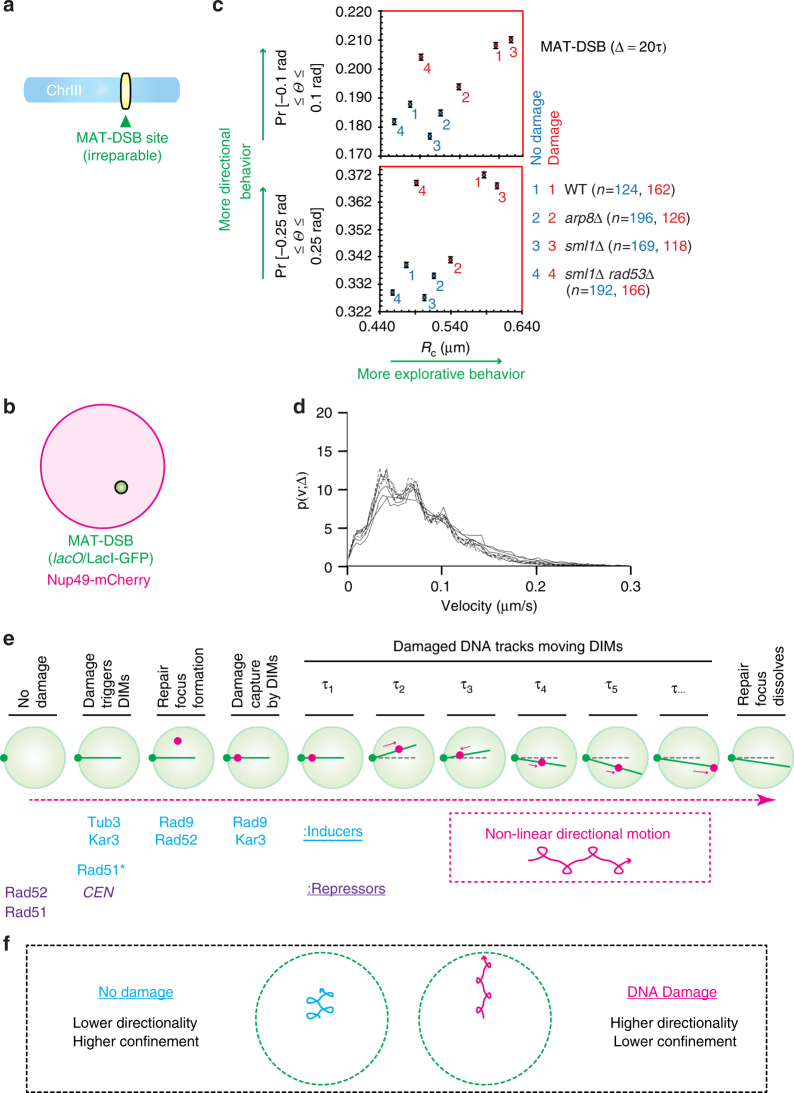
Damage increases the directional motion of DNA. **a**, **b** MAT-DSB schematic (**a**) and system used to visualize it relative to the nuclear periphery (**b**). **c** For a single MAT-DSB locus that is imaged under DNA damaging or non-damaging conditions, the DCD-derived probabilities of the locus moving with up to 0.1 or 0.25 rad angle (*Θ*) deviation from a straight line were plotted relative to MSD-derived *R*_c_ values. Error bars, DCD probability errors. **d** DCD-derived distributions of the velocities of the MAT-DSB locus under damaging and non-damaging conditions for the four cell types shown in **c**. Symbols are not shown as no difference was observed between the eight distributions. **e** DIM-dependent non-linear directional motion of damaged DNA in nuclear space. *Effect seen for MMS but not BIR-DSB. **f** Directionality and subnuclear exploration of intact and damaged DNA

Next, we aimed to compare the directionality of a DNA locus before and after its subjection to a DSB at the cell population level. To avoid potentially repressive effects of repair completion on the detection of DSB directionality, we used an inducible and irreparable DSB at the *MAT* locus on chromosome III (MAT-DSB) (Fig. 4a, b)^25^. Efficient targeting of the induced MAT-DSB to the nuclear periphery is dependent on the Arp8 subunit of the INO chromatin remodeling complex and the Rad53 DNA damage checkpoint protein^5,25,46,47^. Thus, we asked how damage induction alters the directional and explorative behaviors of MAT-DSB in wild-type cells as well as in cells lacking Arp8 or Rad53. Interestingly, at Δ = 20*τ*, the marked DNA locus exhibited directional behavior even before damage induction (Supplementary Fig. 4a). Triggering DNA damage induced DIMs and increased directional and explorative behaviors (Fig. 4c; Supplementary Fig. 4a, b). These increases in directional motion and nuclear exploration were both blunted in *arp8Δ* cells (Fig. 4c). In *rad53∆* cells (the lethality of which was rescued with *sml1Δ*)^5^, DNA damage-induced increases in directionality and exploration were minimally and substantially compromised, respectively (Fig. 4c). Consistent with our data so far (Fig. 3e), DCD-derived distributions of single particle velocities across cell populations also failed to reveal any changes in the velocities of chromatin regardless of DNA damage induction or the loss of Apr8 or Rad53, further supporting the notion that the distribution of directionality and velocity are not necessarily coupled (Fig. 4d). Taken together, our findings indicate that intact DNA exhibits directional motion and subnuclear exploration behaviors that are both increased upon DNA damage. In addition, factors required for increased DNA mobility and repair can promote the directional and/or explorative behavior of damaged DNA.

## Discussion

Our findings reveal that upon DNA damage, loss of centromeric restraint results in the induction of DIMs (Fig. 4e). Damaged DNA is then captured by DIMs inside nuclei in a manner that is dependent on molecular motors and DNA damage response proteins. The captured damaged DNA moves linearly along DIMs towards NPCs before being ultimately resolved (Fig. 4e). However, single live cell imaging revealed that DIMs themselves oscillate inside the nucleus resulting in damaged DNA exhibiting non-linear directionality in nuclear space (Fig. 4e). By adjusting for potential DIM oscillations, novel DCD-based analysis of the mobility of damaged DNA inside the nucleus across the cell population also revealed non-linear directional motions (Fig. 4f). This directionality is decreased upon disruption of DNA repair-promoting factors including molecular motors.

Unexpectedly, despite the increased directionality of damaged DNA, intact DNA still exhibits significant directional behavior. This may be reflective of the observed lower levels of DIMs in the absence of DNA damage induction. Alternatively, this could be reflective of the existence of additional active or passive processes ensuring a baseline level of directional motion in the absence of DNA damage. Consistent with this possibility, we observe that the decreased directionality of damaged DNA in the absence of repair-promoting factors is not lower than the baseline directionality of intact DNA. In addition, the increased linear behavior of damaged DNA generally occurs in a nuclear context that allows for more explorative behavior while the baseline directionality of intact chromatin is more spatially confined.

Importantly, our findings in yeast provide the first mechanism that can resolve the damaged DNA mobility paradox. More specifically, it was hitherto unclear how the cytoplasmic microtubules may cooperate with damaged DNA-interacting nuclear motors, which mediate the directional motion of cargo, to promote an increased yet non-directed mobility of damaged DNA. Our results resolve this paradox by revealing that damaged DNA can move onto proteinaceous nuclear filaments that are themselves moving within nuclear space. The resulting non-linear directional motion is not readily captured by MSD analysis but can be uncovered using DCD analysis^44^. Thus, it will be critical that the abundant single particle motion datasets related to both intact and damaged DNA mobility in the field are re-analyzed using methods combining DCD and MSD. This could reveal that the mobility of various damaged DNA loci is not random as previously thought. Re-analysis may also reveal new functions for various repair-promoting factors. Beyond DNA repair, mobile biological structures whose motion had been previously studied using only MSD analysis should be re-examined using DCD analysis. This should reveal a new layer of complexity in various biological processes.

The mobility of damaged DNA along filamentous proteins inside the nucleus may be evolutionarily conserved. Consistent with this possibility, the formation of intranuclear actin filaments inside mammalian nuclei is required for DNA repair albeit it remains unclear if such filaments directly contribute to an increased DNA mobility and its role in repair^48^. In addition, various mammalian tubulins localize inside the nucleus under standard growth conditions, upon oncogenic transformation or in response to environmental stress^29^. Moreover, molecular motors localize to damaged DNA sites in budding yeast and mammalian nuclei^4,24^.

Our findings reveal how molecular motors and inducible microtubules actively promote damaged DNA mobility and repair. However, relative to random or diffusion mobility, the specific advantage provided by this active transport of damaged DNA in the promotion of DNA repair remains unclear, especially when considered in light of the fact that changes in directionality are not necessarily matched by changes in velocity. For damaged DNA that is targeted to NPCs for repair, one possible advantage is that motors and filaments may guide DNA lesions to specialized types of NPCs^49,50^. Alternatively, docking of the lesions at NPCs may require physical forces that are exerted by microtubules to overcome liquid phase barriers that can block key steps in DNA repair^51^. Similarly, in cases where damaged DNA needs to escape repressive heterochromatin environments, active transport may allow damaged DNA to break through liquid phase barriers separating open and silent chromatin domains^52,53^. Future work should directly test these possibilities.

In closing, DNA damage is very common, damaged DNA exhibits increased mobility in different species, and microtubules or motors promote DNA mobility-dependent repair in yeast and mammalian cells^4–22^. Therefore, the herein identified DIMs and directional DNA motions uncover a missing dimension of DNA repair that widely impacts our understanding of genome organization, cell survival and the movement of biological structures.

## Methods

### Basic strains and materials

Endogenous genes were deleted or modified with C-terminal or N-terminal fluorescent tags. Established protocol for lithium acetate-based yeast transformation was used^4^. All genomic manipulations were confirmed via PCR. Briefly, transformants were screened via PCR using a forward primer that anneals ~200–300 bp upstream of the open reading frame and a reverse primer within the selection marker. Resulting amplicons of the expected size confirmed integration. Successful fluorescent tagging of proteins were further confirmed by live cell confocal microscopy. Yeast strains, plasmids, and primers used in this study are listed in Supplementary Tables 1, 2, and 3.

### Visualization of DIM processes under genotoxic stress

W303 MATa was crossed to KMY2309 to introduce a second genomic copy of N-terminal GFP-tagged *TUB1* under its endogenous promoter. Cells were subsequently transformed with pKM113 (*NOP1-CFP-LEU2-KANMX*)^12^. To allow for Rad52 visualization in subsequent strains, KMY3096 was transformed with pKM198 (*RAD52-YFP-TRP1*). This resulted in the generation of strains KMY3096 and KMY3107.

### Visualization of DIM processes upon single DSB induction

*TetR-GFP-NATMX* was amplified via PCR from pKM271 and integrated under the *URA3* promoter of W303a. The *csURA3csa tetO* × 224 construct was integrated at *YKL222C* by transforming cells with *Bmt*1-linearized pKM255. Orientation of the integrated construct was confirmed via PCR, indicating that the *tetO* array is located internally to the *csURA3csa* construct. The *tetO* repeats and the nearest I-SceI cut site are separated by 2.3 kb of extraneous DNA. Therefore, there is no concern of losing any of the operators to resection as the maximum distance of DSB resection observed is 1.55 kb^54^. The NHEJ-DSB control KMY3323 was generated via transformation with *Eco*NI-linearized pKM250. PCR confirmed that the *csURA3csa* construct is located internally to the *tetO* array at *YKL201C*. For nuclear envelope visualization, endogenous *NUP49* was C-terminally tagged with GFP. For nucleolar imaging strains, *NOP1-CFP-TRP1* under the *NOP1* promoter was integrated at the *LEU2* locus and the full *HIS3* open reading frame was replaced with the *HPHMX* cassette followed by integration of *RAD52-YFP-HIS3* under the *RAD52* promoter at the *TRP1* locus. Cells were then transformed with pKM97 (I-SceI (galactose-inducible), *LEU2d*, *TRP1*)^26^ and either pKM282 (*GFP-TUB1-HIS3*) or pKM334 (*GFP-TUB1-ADE2*). This resulted in the generation of strains KMY3151, KMY3323, and KMY3277.

### Plasmids for generation of *tetO* array-marked DSB sites

pKM255 (BIR-DSB) was generated by first cloning PCR-derived *URA3* flanked by two inverted I-SceI cut sites into pKM217^12^ with *Aat*II and *Nsi*I (primers: URA3-AatII-ISceI-F and URA3-NsiI-ISceI-R). To allow for homology-directed integration, a 1.28 kb fragment of *YKL222C* harboring a unique internal *Bmt*I restriction site was amplified via PCR and cloned into the *csURA3csa tetO* × 224 plasmid using *Sac*I and *Nsi*I (primers: YKL222-NsiI and YKL222-SacI). For integration of the construct into *YKL201C* (NHEJ) and thus generation of pKM250, a 1.78 kb fragment of *YKL201C* containing a unique internal *EcoN*I and flanking *Sac*I and *Nsi*I cut sites was amplified via PCR and cloned into the *csURA3csa tetO* × 224 plasmid (primers: YKL201-F, YKL201-R).

### Live cell imaging

Cells were grown to log phase, pelleted via centrifugation, washed with ddH_2_O, and resuspended in SC media before mounting on a slide for imaging. In asynchronous cell cultures, the small-budded S-phase cells were subjected to microscopy. Images were acquired with a Nikon C2+ Confocal Microscope using a Plan-Apochromat TIRF ×100 oil objective (numerical aperture 1.45) and processed with NIS-Elements AR (Nikon). Imaging was achieved with excitation wavelengths of 405, 488, and 543.5 nm with a 30–40 nm pinhole. Super-resolution time-lapse microscopy under MMS was captured with a Nikon N-SIM E Microscope using an SR-Apochromat TIRF ×100 oil objective (numerical aperture 1.49) and processed with NIS-Elements AR (Nikon). Super-resolution time-lapse microscopy of the BIR-DSB was acquired with the Zeiss Axio Observer.Z1/7 microscope with LSM 800 Airyscan using a Plan-Apochromat ×63 Oil DIC M27 objective (numerical aperture 1.40) and processed with Zen Blue (Zeiss). Signal was detected with an excitation wavelength of 488 nm, and pinhole was 205 µm. For treatment with genotoxic agents, cells were treated with MMS (0.03%), zeocin (50 µg/mL), or camptothecin (5 µg/mL) for 1 h before imaging. For galactose-induced single DSB and CEN3 release, cells were grown on appropriate selection plates with 2% glucose for seven days, then grown to log phase in liquid media with 2% glucose, pelleted via centrifugation, and resuspended in media with 2% galactose to incubate for 1.5 h before imaging. For DSB-DIM capture experiments, only S-phase cells exhibiting both DIMs and Rad52-YFP foci were scored. Spatial overlap of a Rad52-YFP focus with a DIM in a single focal plane for at least 15 s was scored as capture. Spindle and DIM intensity profiles were acquired using the NIS-Elements AR analysis software (Nikon).

### DCD analysis of time-lapse imaging of damaged DNA

DCD analysis was adapted from published work^44^ to the study of damaged DNA mobility as follows. Let the two-dimensional position of a Rad52 focus or DNA locus be denoted by ${\boldsymbol{X}}\left( t \right) = \left[ {x\left( t \right)y\left( t \right)} \right]^{\mathrm{T}}$, where T is the matrix transpose and it is assumed that ***X***(*t*) is sampled at positive integer multiples of the sample period $\tau ,t_k = k\tau ,k = 1,2,3, \ldots$. The average velocity at each sample instant is denoted by $\boldsymbol{V}\left( {t_k;\Delta } \right) = \left[ {{\boldsymbol{X}}\left( {t_k + \Delta } \right) - {\boldsymbol{X}}\left( {t_k} \right)} \right]{\mathrm{/}}\Delta$, where Δ is the temporal coarse-graining parameter which is assumed to be a positive integer multiple of *τ*. The relative angle between the average velocity at *t*_*k*_ and *t*_*k*+1_ for a given Δ is denoted by $\theta \left( {t_k;\Delta } \right)$ and can be determined from the expression for the dot product: ${V}^{\mathrm{T}}\left( {t_{K + 1};\Delta } \right){V}\left( {t_{k};\Delta } \right) = \left| {\boldsymbol{V}\left( {t_{k + 1};\Delta } \right)} \right|\left| {\boldsymbol{V}\left( {t_{k};\Delta } \right)} \right|\,\cos \,\theta \left( {t_{k};\Delta } \right).$This yields a value for $\theta \left( {t_k;\Delta } \right)$) between 0 and *π* where a value of 0 over all times *t*_*k*_ is indicative of straight-line behavior. Next, for a given value of Δ, a histogram of the values of $\theta \left( {t_k;\Delta } \right)$ is constructed across all times in the trajectory, *t*_*k*_, and over all particles (in this work a bin width of 2*π*/100 is used for the histogram). It is normalized to have unit area so as to form the probability density function $\rho \left( {\theta ;\Delta } \right)$. Given the even nature of the cosine function, we set $\rho \left( {2\pi - \theta ;\Delta } \right) = \rho \left( {\theta ;\Delta } \right)$ for $0 \le \theta \le \pi$ with ${\int}_0^{2\pi } {\rho \left( {\theta ;\Delta } \right){\mathrm{d}}\theta } {\mathrm{/}}\left( {2\pi } \right) = 1.$ The probability Pr(·) that the relative angle *θ* lies between −*α* to +*α* can be determined from $I\left( \alpha \right) = \Pr \left[ { - {\mathrm{\alpha }} \le {\mathrm{\theta }} \le {\mathrm{\alpha }}} \right] = {\int}_{ - \alpha }^\alpha {\rho \left( {\theta ;\Delta } \right){\mathrm{d}}\theta } {\mathrm{/}}\left( {2\pi } \right)$, where *θ* = −*α* corresponds to *θ* = 2*π* − *α*. Note that the probability density function and the measure *I*(*α*) serve as measures of directional DNA behavior. For a perfect straight line, the values of $\rho \left( {\theta ;\Delta } \right)$ would cluster near *θ* = 0 and *θ* = 2*π* with *I*(*α*) = 1 for arbitrarily small positive *α*. For erratic DNA motion over small time intervals and using small Δ, it is expected that $\rho \left( {\theta ;\Delta } \right)$ will be nearly one overall all angles *θ*. As more average behavior is considered by increasing Δ, the appearance of some peaking near *θ* = 0 and *θ* = 2π will reveal the emergence of directional behavior. Peaking near *θ* = *π* is the antithesis of such motion. The measure *I*(*α*) for a given *α* gives us a way to compare the degree of directional behavior of DNA in cells with different genotypes. Shown are the significant digits of the calculated probability values.

New findings presented in Figs. 3c, d, 4c, d and Supplementary Figs. 3c and 4a were generated via the above-described novel DCD-based analysis of raw data previously analyzed by MSD in our prior publications^4,5^. Nuclear alignment was performed using Nup49-mCherry images in Mathworks while particles were tracked with the SpotTracker plugin in ImageJ. Available tracking coordinates (*x*, *y*, time) were subjected to DCD-based analysis with no additional cell exclusion or tracking criteria. DCD-based analysis of the same raw data previously analyzed by MSD allows for a full understanding of how DCD uncovers a new dimension of information in data sets previously only analyzed by MSD. The raw data had been previously generated^4,5^ using yeast cells expressing Nup49-mCherry and Rad52-YFP. The cells were grown in SD-leu 2% raffinose media overnight with additional selection for plasmids. Galactose was added for 2.5 h to induce a DSB at 3% final concentration. Cells were mounted in a concanavalin A-coated 8-well chamber (LabTek II, Nalge-Nunc) and maintained at 30 °C. Live cell time-lapse microscopy was performed using a DeltaVision Elite (Applied Precision) with a ×100/1.40 NA Plan-Apochromat oil immersion objective (Olympus) and CoolSNAP HQ2 CCD Camera (512 × 512 with 2 × 2 bin, Roper Scientific). Single plane images were acquired in the YFP channel every 1.5 for 180 s (490 nm excitation, 200 ms exp) while mCherry images were captured every fifth frame (575 nm excitation, 200 ms exp). Nuclear alignment was performed using Nup49-mCherry frames in MATLAB while Rad52-YFP was tracked using the SpotTracker plugin in ImageJ to yield *X*,*Y* coordinates^55^. MSD was calculated using MATLAB. The radius of confinement was calculated as *R*_c_= 5/4*$\surd$MSD calculated from the average MSD value of the last 20 time intervals (121.1–150 s).

### DCD error calculations

Let *θ*_*i*_, *i* = 1,…,*N*, denote the calculated value of the angles determined from the position measurements (*x*_*j*_,*y*_*j*_), *j* = 1,…, *M*. The uncertainty on each *x*_*j*_ and *y*_*j*_ is ±200 nm and the corresponding uncertainty on *θ*_*i*_ is ±*ε*_*i*_, where it is assumed that the angle is uniformly distributed on [*θ*_*i*_ − *ε*_*i*_, *θ*_*i*_ + *ε*_*i*_]. Let [−*α*, *α*] denote a range of angles for which a probability of inclusion has been calculated, that is, *I*(*α*) = Pr[−*α* ≤ *θ* ≤ *α*] = *N*_*α*_/*N*, where *N*_*α*_ is the number of calculated angles fitting in the range [−*α*, *α*] and *N* is the total number of measurements. Now, let _*pi*_ denote the probability that *θ*_*i*_ is in the range [−*α*, *α*] (this is determined by integrating the uniform probability distribution between the appropriate limits corresponding to the overlap with [*θ*_*i*_ − *ε*_*i*_, *θ*_*i*_ + *ε*_*i*_]). Hence, _*pi*_ = 1 corresponds to the case where *θ*_*i*_ ± *ε*_*i*_ is guaranteed to lie inside [−*α*, *α*] and _*pi*_ corresponds to 0 when it is guaranteed to lie outside this range. The variance in *N*_*α*_ can be determined from that of the Bernoulli distribution and is given by $\sigma ^2 = \mathop {\sum}\nolimits_{i = 1}^{{N}} {p_{\mathrm{i}}\left( {1 - p_{\mathrm{i}}} \right)}.$ Then, assuming that *N*_*α*_ has a uniform distribution, we can assume it lies in the range [*N*_*α*_ − √3*σ*, *N*_*α*_ + √3*σ*]. The uncertainty on the probability *I*_*α*_ is then ±√3*σ* /*N*.

### MSD curve fitting and MSD *α*-value calculations

Let MSD *=* *<*(*x*(*t* + Δ*t*) − *x*(*t*))^2^>, where *x* is the position of the focus and *t* is time. MSD fitting was achieved using MSD *=* *Γt*^α^, where *Γ* is a generalized coefficient and α is a time-dependence coefficient reflecting the type of single particle mobility. *α* ∼ 1 reflects normal diffusion, *α* < 1 reflects subdiffusion or anomalous diffusion, and *α* ≥ 2 reflects directed mobility.

### BIR-DSB repair efficiency

Experiments were conducted as described with minor modifications^4,26,37^. Cells containing the *URA3* cassette flanked by two inverted I-SceI cut sites at the subtelomere of the left arm of chromosome XI were freshly transformed with the pKM97 plasmid allowing for galactose-inducible I-SceI expression^26^. Repair efficiency was assessed by comparing cell survival on plates containing galactose versus glucose^26^. Experiments consisted of three biological replicates, four technical replicates per condition within each biological replicate.

### BIR-DSB-2 and MAT-DSB systems

For BIR-DSB-2, we employed a system in which an HO-induced break on chromosome V engages donor DNA sequences on chromosome XI to recreate a functional *CAN1* gene while removing an *HPH* resistance gene via BIR-dependent repair^56^. For the MAT-DSB, we employed a system in which an HO-induced break can be triggered at the MAT locus on chromosome III in cells lacking any donor DNA sequences that are needed for repair and typically present at the silent mating type loci *HML* and *HMR*^5,25^.

### Statistical analysis

For angle probabilities, DCD histograms are probability density functions that were used to compute angle deviation probabilities via computation of areas under the curve. For all data, the number of independent experiments and method of statistical analysis is specified in each figure or figure legend. Individual data points related to bar graphs are shown where applicable. For quantified microscopy experiments in Figs. 1 and 2 and Supplementary Figs. 1, 3, and 4, three biological replicates were completed with a sample size of 40 cells per condition per biological replicate, unless otherwise indicated. Sample sizes were selected to be as large as biologically and technically feasible within our experimental conditions. To compare normally distributed data sets, two-tailed *t* tests were used. Two-way analysis of variance (ANOVA) was used to compare mean differences between multiple conditions and mutants. The variance was similar between the groups that are being compared. GraphPad Prism 7 software was used for all standard statistical analyses. Exact *P* values are as follows: In Fig. 1c, no damage versus damage *P* < 0.0001. In Fig. 1d, compared to Vehicle, *P* values are 0.0004 (MMS and CPT) and 0.0012 (ZEO). In Fig. 1f, Vehicle versus MMS *P* = 0.0004. In Fig. 1g, compared to wild-type (WT), *P* values are <0.0001 (*tub3∆, rad52∆, rad9∆, kar3∆, nup84∆, pol32∆*) and 0.4904 (*dpb3∆*). In Fig. 1h, undamaged versus damaged cells, *P* < 0.0001 (WT), >0.9999 (*tub3∆*), 0.0588 (*rad52∆*), 0.0101 (*rad9∆*), >0.9999 (*kar3∆*), and <0.0001 (*nup145∆*). Compared to undamaged WT, undamaged mutant *P* values are >0.9999 (*tub3∆*), 0.0035 (*rad52∆*), >0.9999 (*rad9∆*), >0.9999 (*kar3∆*), and >0.9999 (*nup145∆*). Compared to damaged WT, damaged mutant *P* values are <0.0001 (*tub3∆*), 0.7082 (*rad52∆*), >0.9999 (*rad9∆*), 0.1828 (*kar3∆*), and >0.9999 (*nup145∆*). In Fig. 1i, undamaged versus damaged cells, *P* = 0.002 (WT), >0.9999 (*tub3∆*), 0.1304 (*rad52∆*), 0.0138 (*rad9∆*), 0.998 (*kar3∆*), and <0.0001 (*nup145∆*). Compared to undamaged WT, undamaged mutant *P* values are >0.9999 (*tub3∆*), 0.0011 (*rad52∆*), >0.9999 (*rad9∆*), >0.9999 (*kar3∆*), and >0.9999 (*nup145∆*). Compared to damaged WT, damaged mutant *P* values are <0.0001 (*tub3∆*), 0.3181 (*rad52∆*), >0.9999 (*rad9∆*), 0.2232 (*kar3∆*), and 0.9927 (*nup145∆*). In Fig. 1j, *P* = 0.0007. In Fig. 1k, WT constraint damage versus no damage, *P* = 0.0005. Constraint no damage versus no constraint damage, *P* = 0.0003. Constraint no damage versus damage, *P* = 0.9997. WT constraint versus no constraint damage, *P* = 0.9653. In Fig. 2d, Vehicle versus MMS *P* = 0.0039. In Fig. 2e, compared to WT, *P* values are 0.0011 (*rad9∆*) and 0.0007 (*kar3∆*). In Fig. 2g, control versus ZIP *P* = 0.0007. In Fig. 2h, CTL no damage versus damage *P* = 0.0005, ZIP no damage versus damage *P* = 0.0067, CTL no damage versus ZIP no damage *P* = 0.0003, CTL damage versus ZIP damage *P* = 0.0044. In Supplementary Fig. 1a, *P* = 0.2879. In Supplementary Fig. 1f, BIR-DSB WT no damage versus damage *P* = 0.0048, BIR-DSB *rad51∆* no damage versus damage *P* = 0.1292, BIR-DSB WT no damage versus *rad51*∆ no damage *P* = 0.0101, BIR-DSB WT damage versus *rad51∆* damage *P* = 0.2846, MMS WT no damage versus damage *P* = <0.0001, MMS *rad51∆* no damage versus damage *P* = 0.9898, MMS WT no damage versus *rad51*∆ no damage *P* = 0.0083, MMS WT damage versus *rad51∆* damage *P* = 0.0123. In Supplementary Figs. 3 and 4, *P* = 0.0026 and 0.0031, respectively. Additional statistical information related to degrees of freedom, *t*, and *F* values are in Supplementary Table 4.

### Code availability

Computer codes used are available upon reasonable request.

### Data availability

All relevant data are available upon reasonable request.

## Electronic supplementary material

Supplementary Information

Description of Additional Supplementary Files

Supplementary Movie 1
