# Supplementary material for: Nuclear microtubule filaments mediate non-linear directional motion of chromatin and promote DNA repair
Source: Nat Commun. 2018 Jul 2;9:2567. doi: 10.1038/s41467-018-05009-7 (PMC6028458; doi:10.1038/s41467-018-05009-7)
Supplement: Supplementary file 1 — Supplementary Information [file 41467_2018_5009_MOESM1_ESM.pdf]

Supplementary Information for

Nuclear microtubule filaments mediate non-linear directional motion of chromatin and promote DNA repair

Oshidari *et al.*

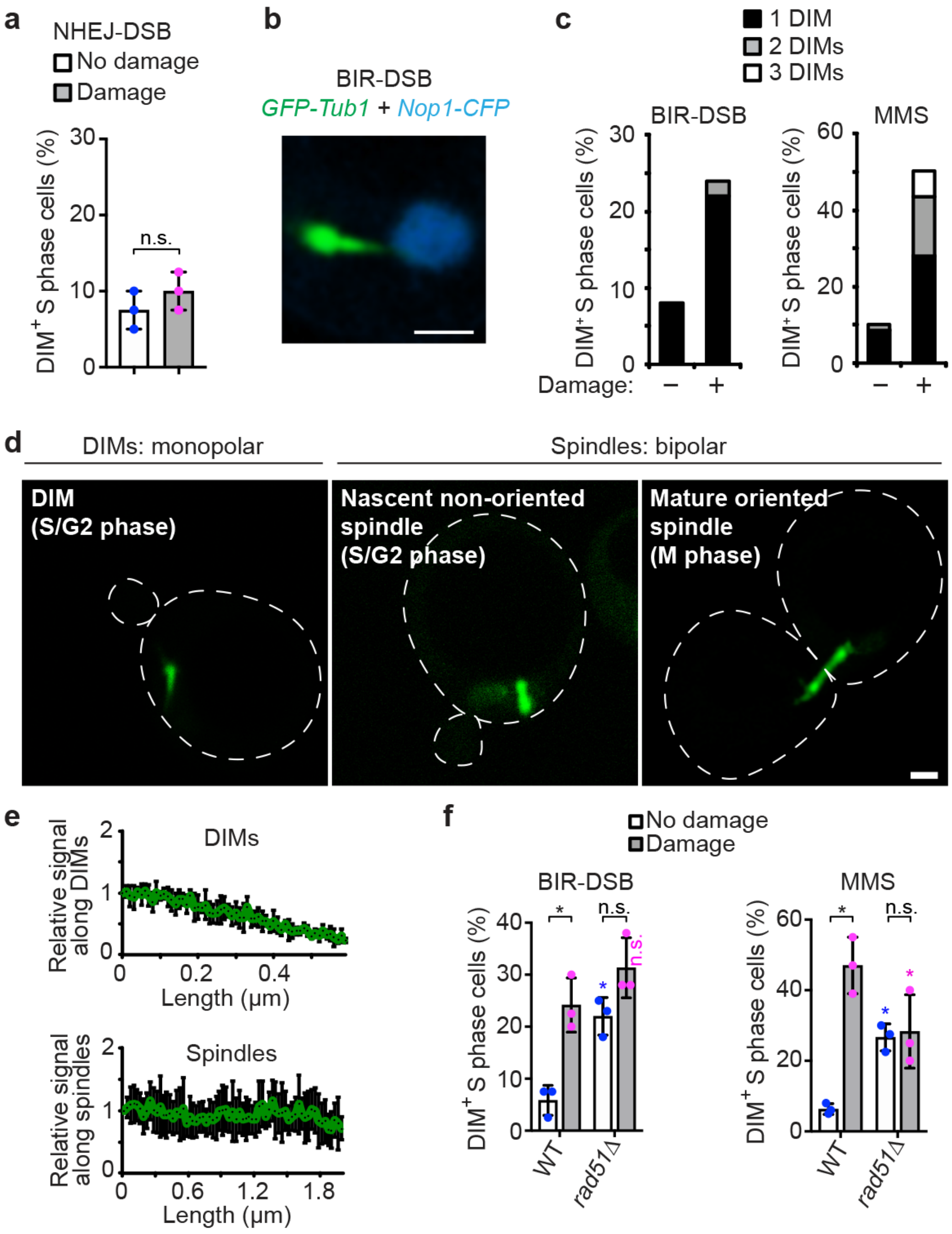

Supplementary Figure 1. Controls related to the reporter systems used and further characterization of DIMs. **a,** Induction of NHEJ-DSB does not significantly induce intranuclear microtubule filaments. Mean ± s.d.; *N*=3. n.s., not statistically significant, two-tailed unpaired *t*-test. **b,** Example of GFP-Tub1 signal in the presence of nucleolar Nop1-CFP expression but in the absence of Nup49-GFP. **c,** Quantification of the number of

DIMs/cell in cell populations subjected to BIR-DSB induction of MMS treatment. Cells are the same as those from **Fig. 1h-i. d,** Representative images for a DIM, nascent mitotic spindle, and mature mitotic spindle. Scale bar, 1 µm. **e,** Measurements of fluorescence intensities across 10 DIMs (top) or 10 spindles (bottom) of various lengths. **f,** Effect of *RAD51* deletion on DIM formation. Mean ± s.d.; N=3. *P≤0.012, 2-way ANOVA Sidak's multiple comparison test. Statistical symbols in blue and magenta indicate a comparison to undamaged and damaged WT control, respectively. Individual data points of undamaged (blue) and damaged (magenta) cells are shown.

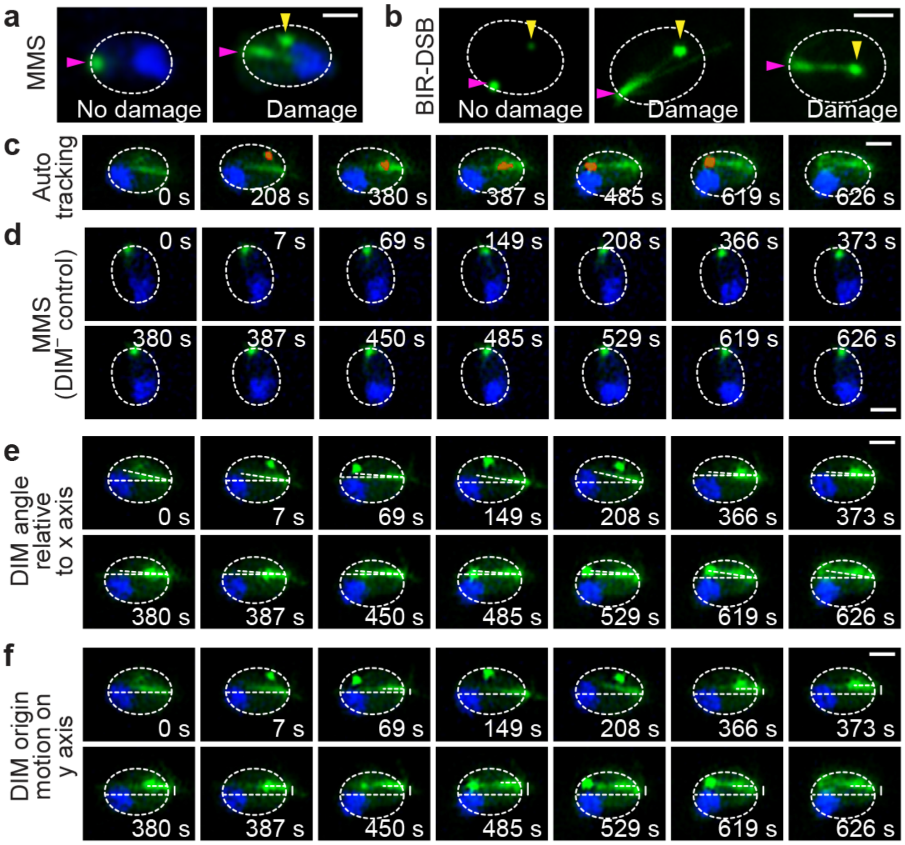

Supplementary Figure 2. Controls and measurement methods related to the tracking of DIM and damaged DNA dynamics. **a,** Representative images of the cell system used to track Rad52-YFP-marked MMS-damaged DNA (yellow arrowhead) relative to GFP-Tub1 (magenta arrowhead) and nucleolar Nop1-CFP (blue) in the presence or absence of DNA damage. **b**, Representative images of the cell system used to track the *tetO*/TetI-marked BIR-DSB site before (yellow arrowhead) and after (yellow arrowhead) DSB induction in cells expressing GFP-Tub1 (magenta arrowhead). **c,** Rad52-YFP foci (shown in Fig. 2b) were auto-tracked using NIS Elements software. **d,** MMS-treated DIM-negative control cell exhibiting the perinuclear MTOC but no Rad52-YFP focus. **e-f**, Representative example of the angular movement of DIMs (**e**) and the perinuclear movement of a DIM's

MTOC origin (**f**, angles are shown here on images also shown in Fig. 2b). **a-f,** scale bar, 1 μm.

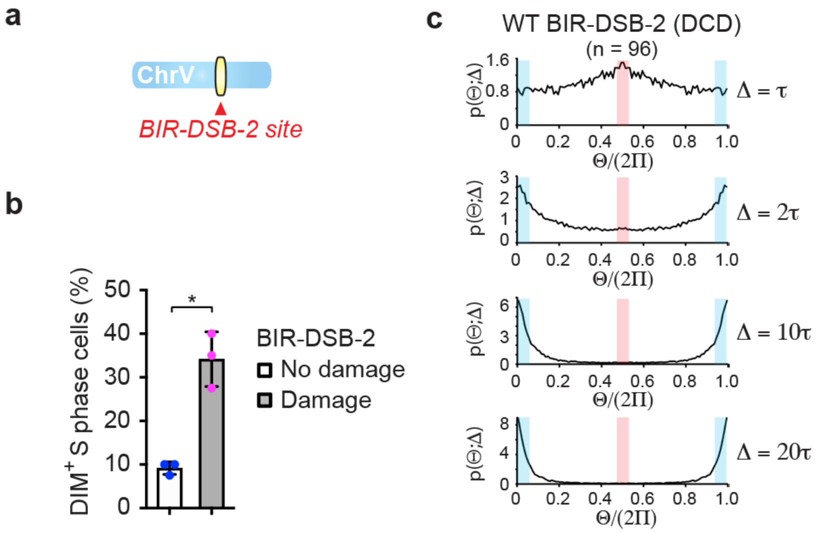

Supplementary Figure 3. BIR-DSB-2 triggers DIMs and exhibits directional motion. **a,** BIR-DSB-2 schematic. **b,** BIR-DSB-2 induction triggers DIMs. Mean ± s.d.; N=3. *P=0.0026, two-tailed unpaired t-test. Individual data points of undamaged (blue) and damaged (magenta) cells are shown. **c,** DCD analysis of damaged DNA mobility reveals non-linear directionality. Shown are relative angle distributions with indicated temporal coarse-grainings (Δ) for a single BIR-DSB-2 across the cell population. Histograms are for particles monitored for 3 min using 1.5 sec long steps. These cells are the same wild-type cells analyzed in Fig. 3d.

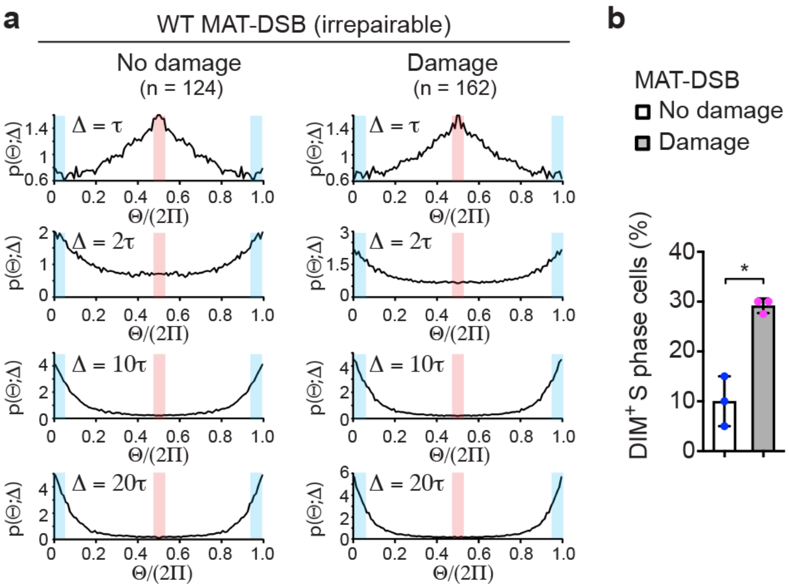

Supplementary Figure 4. DCD analysis of MAT-DSB mobility in wild-type cells. **a,** Shown are relative angle distributions with indicated temporal coarse-grainings (Δ) for an uninduced or induced MAT-DSB locus. Histograms are for particles monitored for 3 min using 1.5 sec long steps. These cells are the same wild-type cells analyzed in Fig. 4c-d. **b,** MAT-DSB induction triggers DIMs. Mean ± s.d.; N=3. *P=0.003, two-tailed unpaired t-test. Individual data points of undamaged (blue) and damaged (magenta) cells are shown.

Supplementary Table 1. List of strains used in this study

KMY#	Genotype
KMY3151	W303 MATa, ura3::TetR-GFP NatMX, YKL222c::csURA3csa TetO x 224 , NUP49-GFP-KANMX, pKM282 (GFP-TUB1-HIS3), pKM97 [I-SceI (gal induc), LEU2d, TRP1]
KMY3096	MATa, ade2-1 trp1-1 can1-100 leu2-3, GFP-TUB1-HIS3, pKM113 (NOP1-CFP-LEU2- KANMX)
KMY3234	KMY3151 SPC42-tdimer2-HPHMX
KMY2357	MATα, ura3 leu2 lys2 GAL, his3::HIS3:GFP-TUB1, pKM113 (NOP1-CFP-LEU2-KANMX)
KMY3324	MATa, ura3-Δ851, leu2Δ1, his3Δ200, lys2Δ202, ykl222c::csURA3csa, pKM97
KMY3325	KMY3324 tub3Δ::HPHMX
KMY3326	KMY3324 rad52Δ::KANMX
KMY3327	KMY3324 rad9Δ::NATMX
KMY3329	KMY3324 kar3Δ::HPHMX
KMY3330	KMY3324 nup84Δ::KANMX
KMY3331	KMY3324 pol32Δ::HPHMX
KMY3332	KMY3324 dpb3Δ::HPHMX
KMY3180	KMY3151 tub3Δ::HPHMX
KMY3179	KMY3151 rad52Δ::HPHMX
KMY3207	KMY3151 rad9Δ::HPHMX
KMY3276	KMY3151 kar3Δ::HPHMX
KMY3280	KMY3151 nup145cΔ::HPHMX
KMY 3350	KMY3151 rad51Δ::HPHMX
KMY3114	KMY3096 tub3Δ::KANMX
KMY3119	KMY3096 rad52Δ::KANMX
KMY3306	KMY3096 rad9Δ::HPHMX
KMY3184	KMY3096 kar3Δ::KANMX
KMY 3351	KMY3096 fob1Δ::KANMX
KMY3354	KMY 3096 rad51Δ::KANMX
KMY3281	KMY3096 nup145cΔ::HPHMX
KMY3252	KMY3096 GAL-CEN3::KANMX
KMY3255	KMY3151 GAL-CEN3::KANMX
KMY3107	KMY3096 pKM198 (RAD52-YFP-TRP)
KMY3277	W303 MATa URA3::TetR-GFP-NATMX, YKL222c::csURA3csa TetO x 224, NOP1-CFP-TRP1, his3Δ:: HPHMX, RAD52-YFP-HIS3, pKM334 (GFP-TUB1-ADE2), pKM97
KMY3313	KMY3306 pKM198 (Rad52-YFP-TRP1)
KMY3316	KMY3276 pKM198 (Rad52-YFP-TRP1)
KMY3220	MATa, ura3-Δ851, leu2Δ1, his3Δ200, lys2Δ202, ykl222c::csURA3csa, cMRS-DSB-L- NATMX (Control sequence), pKM97, NUP49-GFP-KANMX, pKM282 (GFP-TUB1-HIS3)
KMY3222	MATa, ura3-Δ851, leu2Δ1, his3Δ200, lys2Δ202, ykl222c::csURA3csa, MRS-DSB-L- NATMX (Zip sequence), pKM97, NUP49-GFP-KANMX, pKM282 (GFP-TUB1-HIS3)
DDY4302	MATa, ura3-Δ851, leu2Δ1, his3Δ200, lys2Δ202, ykl222c::csURA3csa, pKM97, NUP49-mcherry-HPHMX, pRS413-RAD52-YFP
DDY4296	(Modified JRL346) Mata::HOcsDEL::hisG ura3 DEL851 trp1DEL63 sup53DEL::leu2DEL::NATMX hmlDEL::hisG hmrDEL::ADE3 ade3::GAL10::HO can1,1-1446::HOcs::HPH::DEL AVT2 ykl215c::leu2::hisG::can1DEL1-289, NUP49-mCherry-URA3, pRS415-RAD52-YFP
DDY4298	DDY4296 kar3Δ::KANMX
DDY4299	DDY4298 pRS414-KAR3
DDY4300	DDY4298 pRS414-kar3-1
DDY3165	(Modified JKM179) MATα Δhml::ADE1 Δhmr::ADE1 ade1-110 leu2-3, 112 lys5trp1::hisG, ura3::CUP1-GFP-LAC1-URA3, NUP49-mcherry-HPHMX, ARS313-LACOR-TRP1, ade3::GAL:HO
DDY4117	DDY3165 sml1::NATMX
DDY4121	DDY3165 sml1::NATMX rad53::KANMX
DDY4142	DDY3165 arp8::NATMX
KMY3323	W303 MATa, URA3::TetR-GFP NATMX, YKL201c::csURA3csa TetO x 224 , NUP49-GFP-KANMX, pKM282 (GFP-TUB1-HIS3), pKM97
KMY3320	(Modified JRL346) Mata::HOcsDEL::hisG ura3 DEL851 trp1DEL63, sup53DEL::leu2DEL::NATMX, hmlDEL::hisG, hmrDEL::ADE3, ade3::GAL10::HO ,can1,1-1446::HOcs::HPH::DEL AVT2 ykl215c::leu2::hisG::can1DEL1-289, pKM190 (GFP-TUB1-URA3), pKM113 (NOP1-CFP-LEU2-KANMX)
KMY3333	(Modified JKM179) NUP49-GFP-NATMX, pKM223 (GFP-TUB1-LEU2)

Supplementary Table 2. List of plasmids used in this study.

pKM#	Features	Origin
pKM97	I-SceI (Gal-inducible) TRP1, LEU2	pPEX14
pKM113	NOP1-CFP-LEU2-KANMX	pWJ1529
pKM217	TetOx224-URA3	pWJ1378
pKM271	TetR-GFP-NATMX	This study
pKM190	GFP-TUB1-URA3	Gift from Grant Brown
pKM334	GFP-TUB1-ADE2	This study
pKM223	GFP-TUB1-LEU2	This study
pKM282	GFP-TUB1-HIS3	This study
pKM198	RAD52-YFP-TRP1	Gift from Daniel Durocher
pKM195	RAD52-YFP-HIS3	Gift from Daniel Durocher
pKM250	TetO x 224 csURA3csa YKL201	This study
pKM255	TetO x 224 csURA3csa YKL222	This study

Supplementary Table 3. List of primers used in this study.

Name	Sequence (5'-3')
URA3-AatII-ISceI-F	ATTCGTGACGTCTAGGGATAACAGGGTAATCACGCTTT TCAATTCAATTCATC
URA3-NsiI-ISceI-R	ACGAATATGCATTAGGGATAACAGGGTAATTTAGTTTTGCTGGCCGCATCTTC
YKL222-NsiI-F	ACGAATATGCATATGAAGAATACGGAACTTAGTC
YKL222-SacI-R	ACGAATGAGCTCCCTCGATGATGAGTGGAATG
YKL201-F	CCAGTCCGCTTTCGACAAAG
YKL201-R	CTGGGGATGTTTGGATACCATG

Supplementary Table 4. Full statistical details for all figures.

**Fig. 1c - Two-tailed unpaired t-test**			
Comparison	P Value	t value	Degrees of freedom
No damage vs damage	<0.0001	13.62	44
**Fig.1d - Two-tailed unpaired t-tests**			
Comparison	P Value	t value	Degrees of freedom
Veh vs MMS	0.0004	10.85	4
Veh vs CPT	0.0004	10.96	4
Veh vs ZEO	0.0012	8.246	4
**Fig. 1f - Two-tailed unpaired t-test**			
Comparison	P Value	t value	Degrees of freedom
Veh vs MMS	0.0004	3.619	251
**Fig. 1g - Two-tailed unpaired t-tests**			
Comparison	P Value	t value	Degrees of freedom
WT vs tub3Δ	<0.0001	9.938	20
WT vs rad52Δ	<0.0001	11.94	20
WT vs rad9Δ	<0.0001	11.6	20
WT vs kar3Δ	<0.0001	12.12	20
WT vs nup84Δ	<0.0001	10.17	20
WT vs pol32Δ	<0.0001	11.97	20
WT vs dpb3Δ	0.4904	0.703	20
**Fig. 1h - 2-way ANOVA Sidak's multiple comparison test**			
Comparison	P Value	F value	Degrees of freedom
WT no damage vs WT damage	<0.0001	6.662	(6,28)
tub3Δ no damage vs tub3Δdamage	>0.9999		
WT no damage vs tub3Δ no damage	>0.9999		
WT damage vs tub3Δ damage	<0.0001		
rad52Δ no damage vs rad52Δ damage	0.0588		
WT no damage vs rad52Δ no damage	0.0035		
WT damage vs rad52Δ damage	0.7082		
rad9Δ no damage vs rad9Δ damage	0.0101		
WT no damage vs rad9Δ no damage	>0.9999		
WT damage vs rad9Δdamage	>0.9999		
kar3Δ no damage vs kar3Δ damage	>0.9999		
WT no damage vs kar3Δ no damage	>0.9999		
WT damage vs kar3Δ damage	0.1828		
nup145Δ no damage vs nup145Δ damage	<0.0001		
WT no damage vs nup145Δ no damage	>0.9999		
WT damage vs nup145Δ damage	>0.9999		
**Fig. 1i - 2-way ANOVA Sidak's multiple comparison test**			
Comparison	P Value	F value	Degrees of freedom
WT no damage vs WT damage	0.0002	7.572	(7,32)
tub3Δ no damage vs tub3Δdamage	>0.9999		
WT no damage vs tub3Δ no damage	>0.9999		
WT damage vs tub3Δ damage	<0.0001		
rad52Δ no damage vs rad52Δ damage	0.1304		
WT no damage vs rad52Δ no damage	0.0011		
WT damage vs rad52Δ damage	0.3181		
rad9Δ no damage vs rad9Δ damage	0.0138		
WT no damage vs rad9Δ no damage	>0.9999		
WT damage vs rad9Δdamage	>0.9999		
kar3Δ no damage vs kar3Δ damage	0.998		
WT no damage vs kar3Δ no damage	>0.9999		
WT damage vs kar3Δ damage	0.2232		
nup145Δ no damage vs nup145Δ damage	<0.0001		
WT no damage vs nup145Δ no damage	>0.9999		
WT damage vs nup145Δ damage	0.9927		

**Fig. 1j - Two-tailed unpaired t-tests**			
Comparison	P Value	t value	Degrees of freedom
CEN3 constraint vs no constraint	0.0007	9.499	4
**Fig. 1k - 2-way ANOVA Sidak's multiple comparison test**			
Comparison	P Value	F value	Degrees of freedom
WT Constraint- No damage vs damage	0.0005	0.125	(1,8)
Constraint no damage vs no constraint damage	0.0003		
Constraint- No damage vs no damage	0.9997		
WT constraint damage vs no constraint damage	0.9653		
**Fig. 2d - Two-tailed unpaired t-test**			
Comparison	P Value	t value	Degrees of freedom
Veh vs MMS	0.0039	5.982	4
**Fig. 2e - Two-tailed unpaired t-tests**			
Comparison	P Value	t value	Degrees of freedom
WT vs rad9Δ	0.0011	8.39	4
WT vs kar3Δ	0.0007	9.458	4
**Fig. 2g - Two-tailed unpaired t-test**			
Comparison	P Value	t value	Degrees of freedom
CTL vs ZIP	0.0007	9.533	4
**Fig. 2h - 2-way ANOVA Sidak's multiple comparison test**			
Comparison	P Value	F value	Degrees of freedom
CTL no damage vs damage	0.0005	0.777	(1,16)
ZIP no damage vs damage	0.0067		
CTL no damage vs ZIP no damage	0.0003		
CTL damage vs ZIP damage	0.0044		
**Supplementary Fig. 1a - Two-tailed unpaired t-test**			
Comparison	P Value	t value	Degrees of freedom
No damage vs damage	0.2879	1.225	4
**Supplementary Fig. 1f - 2-way ANOVA Sidak's multiple comparison test**			
Comparison	P Value	F value	Degrees of freedom
BIR-DSB WT no damage vs damage	0.0048	2.972	(1,8)
BIR-DSB rad51Δ no damage vs damage	0.1292		
BIR-DSB WT no damage vs rad51Δ no damage	0.0101		
BIR-DSB WT damage vs rad51Δ damage	0.2846		
MMS WT no damage vs damage	<0.0001	28.25	(1,14)
MMS rad51Δ no damage vs damage	0.9898		
MMS WT no damage vs rad51Δ no damage	0.0083		
MMS WT damage vs rad51Δ damage	0.0123		
**Supplementary Fig. 3 - Two-tailed unpaired t-test**			
Comparison	P Value	t value	Degrees of freedom
No damage vs damage	0.0026	6.708	4
**Supplementary Fig. 4 - Two-tailed unpaired t-test**			
Comparison	P Value	t value	Degrees of freedom
No damage vs damage	0.0031	6.379	4
