# Supplementary material for: Nuclear microtubule filaments mediate non-linear directional motion of chromatin and promote DNA repair
Source: Nat Commun. 2018 Jul 2;9:2567. doi: 10.1038/s41467-018-05009-7 (PMC6028458; doi:10.1038/s41467-018-05009-7)
Supplement: Supplementary file 2 — Description of Additional Supplementary Files [file 41467_2018_5009_MOESM2_ESM.pdf]

Description of Additional Supplementary Files

File Name: Supplementary Movie 1

Description: *Simultaneous visualization of the dynamics of damaged DNA loci and DIMs.* Cells expressing the microtubule marker Tub1-GFP, nucleolar Nop1-CFP and the damaged DNA locus marking Rad52-YFP were subjected to the DNA-damaging agent MMS and imaged using live cell super-resolution microscopy employing the structured illumination microscopy (SIM). Shown is a region of interest with three cells. The cell at the top represents a control cell in which only the MTOC focus is visible while the DIM and Rad52 focus are not formed. In the cell on the right, a Rad52-YFP focus can be observed moving randomly around the nucleus before getting captured by and moving along a DIM towards the nuclear periphery. The cell at the bottom serves as a control in which the Rad52-YFP focus is formed but never captured by an existing DIM.
